# Chronic Complex elbow fracture dislocation: Restoration of elbow function with ORIF and radial head replacement, a case report with long term follow up

**DOI:** 10.1016/j.ijscr.2023.108912

**Published:** 2023-10-03

**Authors:** Eman Faqih, Hasan sawan, Shoog Fahad Alfadhel, Khaled AlAbbasi, Mustafa Alrawi

**Affiliations:** King Fahad Medical City, Riyadh, Saudi Arabia

**Keywords:** Chronic elbow dislocation, Polytrauma, Total elbow arthroplasty, Traumatic arthritis, Terrible triad injury of the elbow

## Abstract

**Introduction:**

The dislocation of the elbow joint to the posterior or postero-lateral region accompanied by fractures in the radial head or neck and coronoid process of the Ulna is known as a terrible triad injury of the elbow (TTI).

**Importance:**

This injury presents as unique challenge for orthopedic surgeons due to elbow instability and stiffness, making the surgical intervention more difficult than usual.

**Case presentation:**

A 47-year-old man suffered from polytrauma, including a pelvis fracture, a left humerus shaft fracture, and left ulna shaft fracture. An open reduction and internal fixation were administered as a treatment option. However, during a follow-up examination four months later, a missed fracture dislocation of the right elbow was discovered.

**Clinical discussion:**

The complex surgery required open reduction of the chronic dislocated joint, release of the triceps, resection of the radial head, replacement, bone grafting of the coronoid, reconstruction of the coronoid, and application of a spanning external fixation.

The injury was complex, consisting of coronoid fractures, olecranon, a proximal third of the Ulna, and radial head malunion with heterotrophic ossification around the elbow joint.

**Conclusion:**

After seven years, our patient had a full range of motion in elbow flexion with 20–25 lags in extension. The Mayo Elbow Performance Score (MEPS) was 100 and Disabilities of Arm, Shoulder and Hand (DASH) score was 0.

## Highlights

• To monitor the long term outcome of the chronic dislocation
• Improve the quality of life after chronic joint dislocation
• Managed missed or late presentation of the dislocated elbow

## 1 Introduction

An elbow fracture is rare, and of those fractures, 36 % result in a radial head fracture. Coronoid process fractures and olecranon fractures are less common, with rates of 13 % and 4 %, respectively. Combining all of these fractures with a “terrible triad” is not well-documented in the literature [1]. In a study by Muthu et al., a case was described of a 40-year-old woman with a complex elbow fracture [1]. Patients with a neglected terrible triad injury are unlikely to fully regain functional use of the elbow. As a result, surgeons face challenges when performing elbow surgery, particularly due to post-traumatic elbow instability. Total elbow arthroplasty (TEA) is a significant surgery for managing a painful, arthritic elbow joint. Another study found that a 50 % loss of the coronoid process did not improve stability with either LCL repair or radial head replacement [2]. Choskey et al. also found that fractures affecting more than 50 % of the coronoid process can lead to axial instability [3].

Treating these fractures can be quite difficult due to the complex anatomy of acute dislocations with significant fractures and joint biomechanics. Using internal fixation to achieve congruity may result in stiffness and loss of functionality, making surgery the only practical solution for TEA with chronic instability. In this report, we present the results of salvage surgery with a 7-year follow-up after the patient ignored TTI. Additionally, the authors provide a clear explanation of the functional reasoning for using a Coonrad-Morrey prosthesis in total elbow replacement for fractures.

## 2 Case presentation

**Fig. 1:**
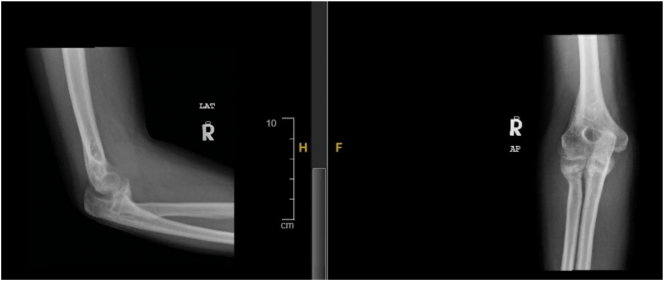
Represents the elbow of the patient showing dislocated joints. X-ray showing dislocation of the right elbow at the first presentation.

In January 2015, a 45-year-old man working as teacher came to the orthopedic clinic complaining of pain, chronic instability, and deformity in his right elbow caused by a vehicle accident four months prior. The injury was complex, involving a right elbow fracture dislocation, a pelvis fracture, a left humerus shaft fracture, and a left ulna shaft fracture. The patient had what is known as a “terrible triad” of the elbow, which included a comminuted radius neck fracture, an impacted radial head fracture, a comminuted coronoid process fracture, and dislocation of the radio-humeral joint. Additionally, there were osteochondral fractures of the trochlea, elbow capsule, annular ligament of radius, and lateral and medial collateral ligaments. These ligamentous tissue injuries caused the elbow joint to be grossly unstable (see Fig. 1), but there was no damage to the nerves or blood vessels. Initially, the range of motion was limited to 0–35 degrees and the movements were unstable, with internal rotation of the elbow when attempting to flex it. The patient's pain score was seven out of 10. The clinical treatment plan included open reduction of the dislocated elbow with radial head replacement, coronoid process fixation, bone grafting, and the application of external fixation to improve the function of the left upper limb after counseling.

## 3 Surgical management

The patient was placed under general anesthesia in a semi-lateral position. The posterior approach was used, with fascio-cutaneous flaps raised to expose the Kocher interval (anconeus and extensor carpi ulnaris). The ulnar nerve was identified, protected, and moved forward into the subcutaneous tissue. The elbow joints were exposed using the triceps-sparing technique. The lateral collateral ligament, which had become detached from the humeral attachment and the overlying musculotendinous envelope, had healed in a displaced position posterior to the epicondyles. The ligament and musculotendinous envelope were mobilized. Next, the radial head was removed. Attention was then directed toward reconstructing the coronoid fracture with bony pieces taken from the radial head as a bone graft. These were then fixed using two screws.

The elbow was secured using a hinged external fixator with two 5-mm threaded pins for the humerus and two 4-mm pins for the Ulna. The humeral pins were positioned to avoid injuring the radial nerve or tethering the triceps mechanism, and the soft tissue was protected during pin insertion with a cannulated sleeve. The pins were manually advanced using a T-handled chuck and confirmed with fluoroscopy. To insert the guide pin through the capitellum's and trochlea's central axis, fluoroscopic guidance was used from a lateral-to-medial direction. The starting point was distal to the lateral epicondyle, and the pin exit was anterior and distal to the medial epicondyle, while ensuring that the pin did not advance beyond the medial cortex by 1–2 mm to protect the ulnar nerve. Proper positioning is critical for optimal stability and range of motion during recovery.

**Fig. 2:**
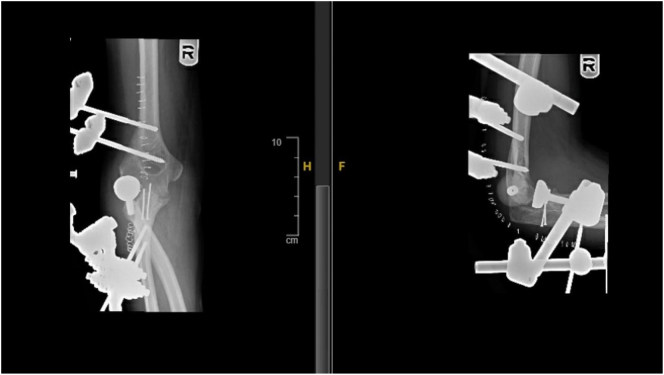
X-ray showing the external fixators and aligning of the radial head prosthesis.

Proper placement of the guide pin is crucial to keep the elbow joint properly aligned throughout the entire range of motion, even after the frame is applied. The articulated external fixator frame was built around the axis guide pin and secured to the pins in the humerus and ulna. The axis guide pin was then removed. To prevent injury from movement, the elbow needed to be immobilized, so an additional locking bar was placed between the ulnar and humeral bars for the first week. Instead of primary reconstruction of the collateral ligaments, the ligaments were repaired with their associated extensor and flexor origins. Suture anchors were used at the lateral and medial epicondyles to approximate the collateral ligaments and muscular origins to the epicondyles. Subcutaneous ulnar nerve transposition was also performed after the external fixator was applied. The wound was closed in layers, and postoperative X-rays showed a well-aligned radial head prosthesis (Fig. 2). The surgery was performed with the knowledge that nerve transposition is beneficial for patients with chronic elbow dislocation who are at a high risk of postoperative ulnar neuropathy.

The hinged external fixator was kept in place for six weeks, during which the locking bar was removed after the first postoperative week. The patient then began a home exercise program with an active range of motion. To ensure proper care, pin-tract maintenance is done twice per day. At the end of the six weeks, the external fixator is removed, and the elbow is checked under general anesthesia. Following this, the elbow is placed into a temporary posterior resting splint for one week. A hinged brace is used for an additional six weeks, while physiotherapy starts the same day. The initial range of motion was between 0 to 35, but it improved to 90 to 30 after the external fixation removal.

**Fig. 3:**
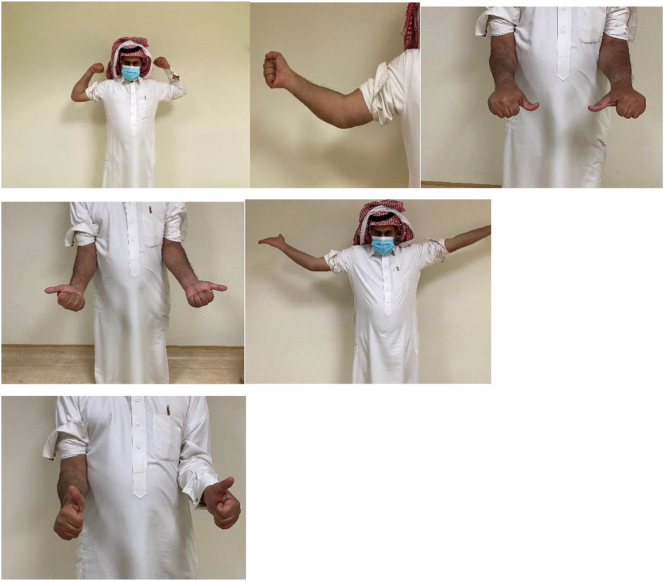
Follow up at 7 years showing good range of motion of the left elbow Figure.

**Fig. 4:**
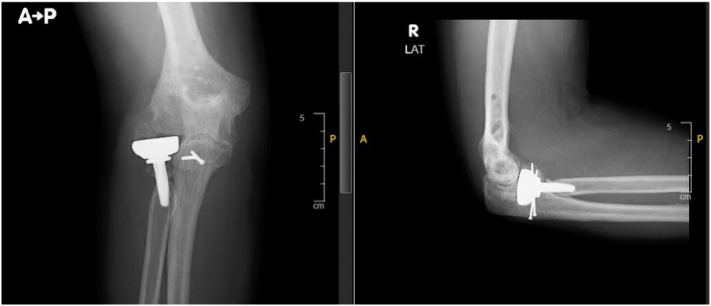
Follow-up X-ray of the right elbow taken after 7 years of the procedures indicating good healing process of the coronoid fracture.

**Fig. 5:**
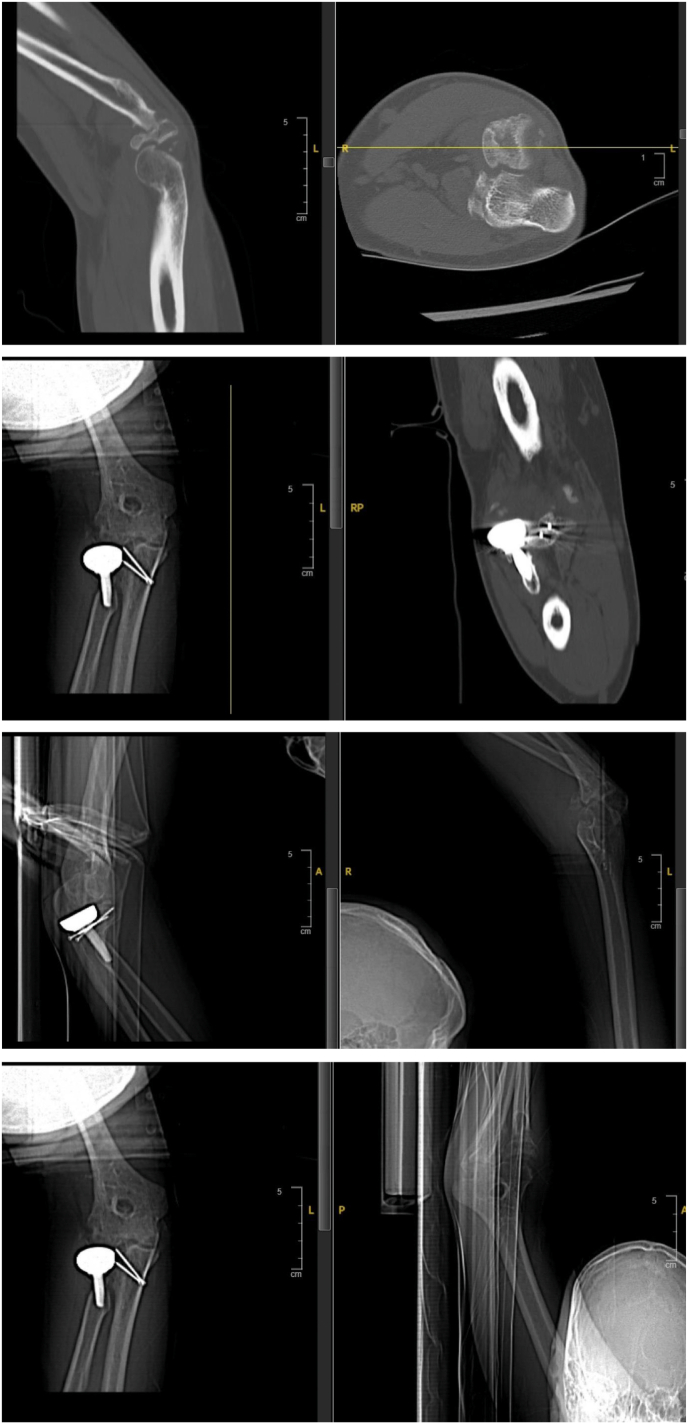
A comparison between elbow CT scan showed pre operative injuries and post operative fixation in axial and sagittal views.

During the six-week postoperative review, the wound had healed, and the patient had gained a range of motion of 90 degrees flexion and 30 degrees lag extension. The patient was also continuously monitored at the clinic. Seven years post-operation, the pain score had improved to 0 out of 10, and the MEPS was 100 and DASH score was 0 (Fig. 3). X-ray imaging showed no osteolysis or loosening of the radial head prosthesis, and a well-healed coronoid fracture (Fig. 4). In comparison with total elbow replacement the chosen of the open reduction with internal fixation was preferred due to his better outcome, shorter period of restore normal elbow range of motion and patient age. Finally, a pre operative and post operative CT scan were done for better pre op planning and to have baseline CT scan for further post op follow up (Fig. 5).

## 4 Discussion

The elbow is the second most commonly dislocated major joint in adults, following the shoulder. Surgery is generally required for less than 10 % of patients and those who undergo surgery have a poor outcome [4]. In the United States, there are an estimated 7000 cases of elbow dislocation annually, with a worldwide incidence rate of 5.21 per 100,000 individuals. However, males have a slightly higher dislocation rate [5]. While posterior and posterolateral dislocations make up 80 % of all cases, lateral, posteromedial, medial, anterior, and divergent dislocations can also occur [6]. A recent study showed that the most frequent cause of elbow trauma (42 %) was slipping onto an outstretched arm. In terms of categorization, 58 % of elbow dislocations were considered challenging while 42 % were simple. Overall, 85 % of patients required surgery, including 73 % who had sustained simple elbow dislocations due to a weak or non-congruent decreased elbow [7].

Chronic dislocation is when a dislocation hasn't been fixed for more than two weeks. This makes it harder to treat because the bones and ligaments change and closed reduction is usually impossible after three weeks. One study found that patients who received treatment earlier had better results in terms of flexion arc achieved after surgery [8]. Many studies have found that patients with chronic elbow dislocation and terrible triad have a consistent pattern of problems, including triceps contracture, collateral ligament contracture, and extensive fibrosis [8,9]. It can also lead to ulnar neuritis and fractures. These types of injuries are rare, and treatment is difficult and usually requires complex surgery [1,8]. There are only a few case reports to support treatment approaches, and there are no large-scale clinical trials. Open reduction is often suggested, and the duration of the dislocation and patient age are important factors in the outcome [10]. Dislocations that last longer than six months usually have too much damage for successful open reduction.

According to several articles, the duration of dislocation is inversely related to postoperative function [2,3,8]. Patients who had surgical reduction for chronic dislocations lasting from one to six months reported no connection between the length of dislocation and postoperative arc of motion. Some studies also showed no difference in the results of patients who underwent surgical reduction between two and six months post-injury [1,7]. Most dislocations occur in the posterolateral region and are commonly linked to fractures. It has been discovered that the medial epicondyle may be trapped in the joint, making initial attempts at closed reduction difficult [11].

Dislocations that cannot be closed often result from improper reduction or insufficient immobilization in the emergency department, combined with delays in referral. These errors can be prevented through patient education and the creation of more specialized clinics in which can patient can be seen early with specialist physician and the best care can be provided. Finally this paper has been reported in line with the SCARE criteria [12].

## 5 Conclusion

In conclusion, our case report indicates that we successfully treated the missed fracture after trauma due to a vehicle accident. The missed fracture involved dislocation of the right elbow, which we treated with open reduction and triceps release, radial head resection, bone grafting, coronoid reconstruction, and external fixation. After surgery, the patient regained full range of motion in flexion and 20–25 lags in extension after seven years. Physiotherapy and exercises were also included in the treatment plan to aid in recovery, as elbow fracture surgery can take several months to heal and requires exercise to regain range of motion. Therefore, surgical treatment with stable anatomical restoration is the most effective approach for complex elbow fracture dislocations. Managing chronic elbow terrible triad injury is a challenge and requires consideration of individual factors such as age, activity level, and goals. Preoperative counseling is necessary, and surgical reduction should be performed step-by-step to anticipate common obstacles and tailor the procedure to each patient. Not all patients are suitable for surgical reduction, and those who are older or have significant articular damage may require salvage procedures. It is recommended to follow medical advice on protection and therapy to avoid long-term complications.

## Informed consent

Written informed consent was obtained from the patient. The study was also approved by the institutional ethics committee.

## Ethical approval

IRB Registration Number with KACST, KSA: H-01-R-012

IRB Registration Number with OHRP/NIH, USA: IRB00010471

Approval Number Federal Wide Assurance NIH, USA: FWA00018774

## Sources of funding

None.

## Author contribution

Eman Faqih

emanafaqih@gmail.com

King Fahad Medical City, Riyadh, Saudi Arabia

Shoog Fahad Alfadhel

shoogalfadhel@gmail.com

King Fahad Medical City, Riyadh, Saudi Arabia

Hasan Sawan

sawan.hasan@gmail.com

King Fahad Medical City, Riyadh, Saudi Arabia

Khaled AlAbbasi

kalabbasi@kfmc.med.sa

King Fahad Medical City, Riyadh, Saudi Arabia

Mustafa Alrawi

mnalrawi@kfmc.med.sa

King Fahad Medical City, Riyadh, Saudi Arabia

## Guarantor

Eman Faqih

Shoog Fahad Alfadhel

## Conflict of interest statement

No conflicts of interest.
